# Outcome of mechanical circulatory support at the University Medical Centre Utrecht

**DOI:** 10.1007/s12471-020-01375-4

**Published:** 2020-02-24

**Authors:** S. E. A. Felix, F. Z. Ramjankhan, M. P. Buijsrogge, K. A. Jacob, F. W. Asselbergs, M. I. F. Oerlemans, J. H. Kirkels, L. W. van Laake, A. M. C. Oppelaar, W. J. L. Suyker, N. de Jonge

**Affiliations:** 1 Department of Cardiology, Division Heart and Lungs, University Medical Centre Utrecht, University of Utrecht, Utrecht, The Netherlands; 2 Department of Cardiothoracic Surgery, University Medical Centre Utrecht, University of Utrecht, Utrecht, The Netherlands; 3 Institute of Health Informatics, Faculty of Population Health Sciences, University College London, London, UK

**Keywords:** Mechanical circulatory support, Outcome, Complications

## Abstract

**Background:**

The prevalence of heart failure (HF) is increasing substantially and, despite improvements in medical therapy, HF still carries a poor prognosis. Mechanical circulatory support (MCS) by a continuous-flow left ventricular assist device (cf-LVAD) improves survival and quality of life in selected patients. This holds especially for the short-term outcome, but experience regarding long-term outcome is growing as the waiting time for heart transplantation is increasing due to the shortage of donor hearts. Here we present our results from the University Medical Centre Utrecht.

**Methods:**

Data of all patients with a cf-LVAD implant between March 2006 and January 2018 were collected. The primary outcome was survival. Secondary outcomes included adverse events defined according to the Interagency Registry for Mechanically Assisted Circulatory Support (INTERMACS) definitions, described per patient year.

**Results:**

A total of 268 patients (69% male, mean age 50 ± 13 years) received a cf-LVAD. After a median follow-up of 542 (interquartile range 205–1044) days, heart transplantation had been performed in 82 (31%) patients, the cf-LVAD had been explanted in 8 (3%) and 71 (26%) had died. Survival at 1, 3 and 5 years was 83%, 72% and 57%, respectively, with heart transplantation, cf-LVAD explantation or death as the end-point. Death was most often caused by neurological complications (31%) or infection (20%). Major bleeding occurred 0.51 times and stroke 0.15 times per patient year.

**Conclusion:**

Not only short-term results but also 5‑year survival after cf-LVAD support demonstrate that MCS is a promising therapy as an extended bridge to heart transplantation. However, the incidence of several major complications still has to be addressed.

**Electronic supplementary material:**

The online version of this article (10.1007/s12471-020-01375-4) contains supplementary material, which is available to authorized users.

## What’s new?

- This is the first study investigating the long-term outcome of mechanical circulatory support (MCS) in The Netherlands.
- The 1‑, 3‑ and 5‑year survival on MCS was 83%, 72% and 57%, respectively. These results support its use as an extended bridge to heart transplantation, as necessitated by the shortage of donor hearts in our country.
- Survival in the period 2006–2012 did not differ from that in 2013–2017.
- Adverse events in terms of major bleeding and stroke occurred 0.51 and 0.15 times per patient year, respectively.

## Background

The prevalence of heart failure (HF) is increasing substantially in Western countries. In the Netherlands already 1.3% of the total population (227,300 patients) suffer from HF. This percentage will certainly grow in the coming decades owing to the aging population and better treatment of heart disease in general [1, 2].

Despite a substantial improvement in prognosis resulting from the use of beta blockers, ACE inhibitors/angiotensin receptor blockers, angiotensin receptor-neprilysin inhibitors, aldosterone antagonists, implantable cardioverter defibrillators and resynchronisation therapy, HF still carries a poor prognosis with a 1-year mortality of 26% in patients below the age of 75 years and 56% for those aged above 75 [3].

In patients with end-stage HF refractory to optimal medical therapy, heart transplantation is ‘the gold standard’ [4, 5]. However, because of the severe shortage of donor hearts, only few patients may benefit from this procedure. It is not to be expected that the number of donor hearts will increase substantially, so alternative treatment options need to be considered. Long-term mechanical circulatory support (MCS) by continuous-flow left ventricular assist devices (cf-LVADs) has demonstrated improved life expectancy and quality of life in these patients and may hold promise for the future as a realistic alternative to heart transplantation [6–10]. On the other hand, management of patients on long-term cf-LVADs is still very laborious owing to well-known adverse events, such as infection, bleeding, thrombosis and device malfunction [11].

In our centre cf-LVADs have been implanted since 2006, initially the HeartMate II (HM-II, Abbott, St. Paul, MN, USA), from 2010 the HVAD (Medtronic, Framingham, MA, USA) and, since the end of 2015, the HeartMate 3 (HM 3, Abbott) [12].

Previously, only relatively short-term results of cf-LVAD implantations in the Netherlands have been published [13, 14]. As the duration of MCS is growing, partly caused by the shortage of donor hearts, this study was performed to provide insight into the long-term outcome in terms of survival and adverse events. Furthermore, we were interested whether the clinical situation of patients before cf-LVAD implantation has changed over the years with respect to Interagency Registry for Mechanically Assisted Circulatory Support (INTERMACS) profile.

## Methods

### Study population

Data of all patients in whom the HM-II, HM 3 or the HVAD was implanted at the University Medical Centre Utrecht between March 2006 and January 2018 (end of study) were collected in a central database. The database included baseline clinical characteristics and all adverse events defined according to the INTERMACS definitions [15]. Adverse events were described for the total population. The institutional ethics board approved the study.

### cf-LVAD implantation and anticoagulation

cf-LVADs were implanted via a median sternotomy using extracorporeal circulation on the beating heart. In the absence of bleeding, heparin was started within 48 h after surgery if drainage during 3 consecutive hours post-implant did not exceed 50 ml/h. A vitamin K antagonist was started after drain removal and heparin was stopped when the INR reached the lower limit of the therapeutic range as described for each cf-LVAD type below.

A thrombocyte aggregation inhibitor was started after 48 h, in general aspirin 100 mg/day. From March 2006 until August 2009, in HM-II patients warfarin was titrated to an INR of 2–2.5, 7 days after implantation. The INR range was reduced to 1.5–2.0 due to a substantial incidence of bleeding, as reported in the literature [16]. From December 2011 the INR range was increased again to 1.8–2.5 because of increased thrombo-embolic complications. For the HVAD and HM 3, target INR was 2.5–3.5 and 2.0–3.0, respectively, according to the manufacturer’s advice.

### Outcome

The primary outcome of the study was survival on cf-LVAD support until a pre-specified end-point, i.e. death, device explantation, heart transplantation or the end of the study. Secondary outcomes included all adverse events defined according to INTERMACS.

### Definition of adverse events

All adverse events were defined according to the INTERMACS definitions. Major bleeding was defined as suspected internal or external bleeding, resulting in death, re-operation, hospitalisation and/or transfusion of red blood cells (within the first 7 days after the implantation requiring transfusion ≥4 units of packed red blood cells, or any transfusion beyond 7 days postoperatively). Neurological complications included a transient ischaemic attack and ischaemic/haemorrhagic strokes. Major infection was defined as a clinical infection accompanied by pain, fever, drainage and/or leukocytosis, treated by antimicrobial agents (non-prophylactic).

Major haemolysis included biochemical signs of haemolysis (free plasma haemoglobin >200 mg/l or lactate dehydrogenase >625 U/l), accompanied by at least one of the following symptoms: haemoglobinuria, anaemia, hyperbilirubinaemia and/or pump malfunction. Minor haemolysis comprised asymptomatic biochemical abnormalities.

Major device malfunction included pump thrombosis, high-urgency transplantation, pump replacement, pump explantation, breach of driveline or death. Minor device malfunction included inadequately functioning external components which required repair or replacement. Right heart failure (RHF) was defined as symptoms and signs of persistent right ventricular dysfunction (central venous pressure >18 mm Hg with a cardiac index <2.3 l/min/m^2^ in the absence of elevated left atrial/pulmonary capillary wedge pressure (>18 mm Hg), tamponade, ventricular arrhythmias or pneumothorax) requiring implantation of a right ventricular assist device, inhaled nitric oxide or inotropic therapy for >1 week at any time after cf-LVAD implantation.

### Statistical analysis

We used SAS software (SAS Institute, Cary, NC, USA) for statistical analysis. Kaplan-Meier survival estimation was applied for survival analysis of the entire cohort, and as specified by INTERMACS profile at baseline. Differences in survival were considered statistically significant if the log-rank test showed a *p*-value <0.05. Comparison of dichotomous variables between implantations from 2006 to 2012 and from 2013 to 2017 was performed by chi-square test or Fisher’s exact test. Continuous variables were compared by the Mann-Whitney U test. In patients who died, the cause of death was retrospectively verified by one researcher and categorised according to the annual INTERMACS reports. Rate of complications was described per patient year.

## Results

### Baseline

From March 2006 until January 2018, 268 patients underwent cf-LVAD implantation (69% male, mean age 50 (±13) years). In 59% of patients HM-II was implanted, 98.5% of devices as a bridge to transplantation, the remaining 1.5% as destination therapy. Follow-up was completed for all 268 patients for a median period of 542 (interquartile range (IQR): 205–1044) days, resulting in a total experience of 510 patient years (mainly determined by 380 patient years for HM-II).

The clinical profile before cf-LVAD implantation was most often INTERMACS 2 (42%) or 3 (27%), implying a progressive decline on inotropic support and stable but inotrope dependent, respectively. Furthermore, 19% of the patients were on temporary MCS prior to cf-LVAD implantation, mostly by central or peripheral extracorporeal life support, so were originally INTERMACS 1 but stabilised on temporary MCS. Baseline characteristics for the total cohort and per device type are summarised in Tab. 1.

**Table 1:** Characteristics of the overall population of patients with a continuous-flow left ventricular assist device (cf-LVAD) as well as per type of cf-LVAD

	Total	HM-II	HVAD	HM 3
	*n* (%)	*n* (%)	*n* (%)	*n* (%)
*Total*	268 (100)	159 (59)	71 (27)	38 (14)
*Age* (years, mean ± SD)	50 ± 13	48 ± 13	54 ± 12	51 ± 14
*Gender—male*	185 (69)	109 (69)	52 (73)	24 (63)
*Aetiology of cardiomyopathy*				
Dilated	146 (54.5)	97 (61)	27 (38)	22 (57.9)
Hypertrophic	5 (1.9)	4 (2.5)	1 (1.4)	0 (0)
Ischaemic	69 (25.7)	35 (22)	27 (38)	7 (18.4)
Myocarditis	11 (4.1)	9 (5.7)	2 (2.8)	0 (0)
Peri-partum	3 (1.1)	2 (1.3)	1 (1.4)	0 (0)
Toxic	7 (2.6)	6 (3.8)	0 (0)	1 (2.6)
Congenital	1 (0.4)	1 (0.6)	0 (0)	0 (0)
Other	26 (9.7)	5 (3.1)	13 (18.3)	8 (21.1)
*INTERMACS profile*				
1 Critical cardiogenic shock without MCS	10 (3.7)	6 (3.8)	3 (4.2)	1 (2.6)
1* Critical cardiogenic shock with MCS	52 (19.4)	24 (15.1)	23 (32.4)	5 (13.2)
2 Progressive decline on inotropic support	112 (41.8)	75 (47.2)	20 (28.2)	17 (44.7)
3 Stable but inotrope dependent	71 (26.5)	43 (27)	18 (25.4)	10 (26.3)
4 Resting symptoms at home on oral therapy	22 (8.2)	10 (6.3)	7 (9.9)	5 (13.2)
6 Exertion limited	1 (0.4)	1 (0.6)	0 (0)	0 (0)

^*HM-II*^ ^HeartMate^ ^II,^^*HM 3*^ ^HeartMate 3,^^*INTERMACS*^ ^Interagency Registry for Mechanically Assisted Circulatory Support,^^*MCS*^ ^mechanical circulatory support^

The INTERMACS profile prior to the cf-LVAD implantation has changed over time. Since 2013, patients in higher INTERMACS profiles received implants more frequently than in the first few years. Statistical analysis was performed to compare survival of patients receiving implants between 2006 and 2012 with those between 2013 and 2017. Patients in whom a cf-LVAD was implanted between 2006 and 2012 were significantly younger (*p* < 0.001) and more frequently in INTERMACS profile 2 (*p* = 0.008) than those receiving implants between 2013 and 2017 (Electronic Supplementary Material, Table 1).

### Primary outcome

Seventy-one (26%) patients (44 HM-II, 24 HVAD and 3 HM 3) died after cf-LVAD implantation after a median of 216 (IQR: 20-807) days. Death was most often caused by neurological complications (22 patients) or infections (14 patients) (Tab. 2). Device malfunction was the cause of death in 7 patients, pump thrombosis in 5 cases and technical failure in 2. Eighty-two (31%) patients underwent a heart transplantation, after a median duration of 674 (IQR: 394–1028) days on cf-LVAD-support. Explantation of the cf-LVAD was possible in 8 (3%) patients after a median period of 529 (IQR: 351–670) days. In these 8 patients, myocarditis and peri-partum cardiomyopathy were the most common aetiologies. In one patient with dilated cardiomyopathy in whom the cf-LVAD was initially explanted following the recovery of left ventricular function, a new device had to be implanted after 144 days due to recurrent HF.

**Table 2:** Cause of death, also divided into peri‐operative and late mortality

Cause of death	Number of patients (%)	≤30 days postoperative	>30 days postoperative
Multi-organ failure	4 (5.6%)	2 (9.5%)	2 (4%)
RV failure	7 (9.9%)	1 (4.8%)	6 (12%)
Device malfunction	7 (9.9%)	0 (0%)	7 (14%)
Neurological	22 (31%)	4 (19%)	18 (36%)
Infection	14 (19.7%)	5 (23.8%)	9 (18%)
Other	17 (23.9%)	9 (42.9%)	8 (16%)
Total mortality	71 (100%)	21 (29.6%)	50 (70.4%)

*RV failure* therapy-refractory right ventricular failure leading to death

Device malfunction includes technical failure of the pump itself and pump thrombosis. Neurological causes of death include haemorrhagic or ischaemic stroke. Infection comprises systemic infections, non-responsive to the applied treatment. Other causes of death include multifactorial and unknown causes of death, for example in patients in whom no autopsy was performed

**Fig. 1:**
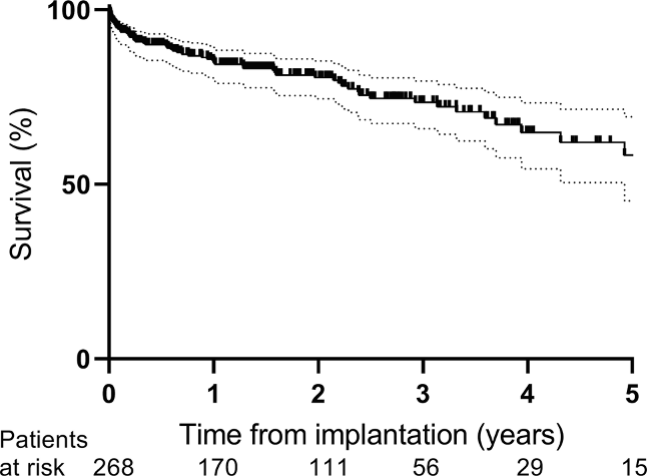
Kaplan Meier survival curve. *Dotted line* 95% confidence interval

**Fig. 2:**
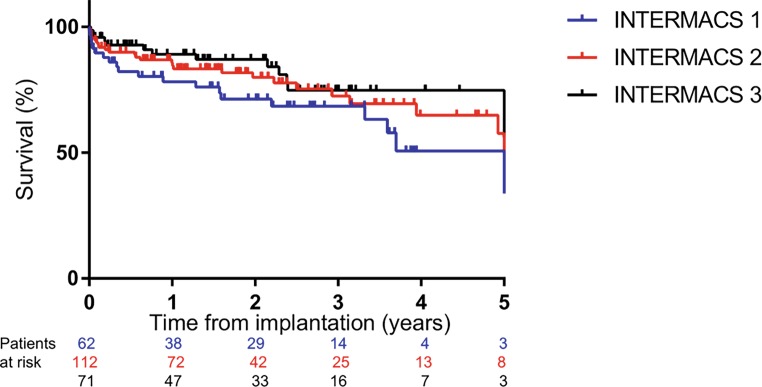
Kaplan Meier survival curve, stratified by INTERMACS profile

Survival after 1, 3 and 5 years was 83%, 72% and 57%, respectively (Fig. 1). There was a trend towards worse survival in patients with INTERMACS profile 1 in comparison to INTERMACS profile 2 or 3, though not significantly different (Fig. 2, *p* = 0.24). Neither did survival differ significantly between implants in 2006–2012 in comparison to implants in 2013–2017 (*p* = 0.44). Because patients receiving implants between 2006 and 2012 were significantly younger and more frequently in INTERMACS 2 in comparison to 2013–2017, correlation between survival and these variables was analysed. Both age and INTERMACS profile 2 were not associated with mortality in this cohort (hazard ratio (HR) 0.98 (95% confidence interval, CI, 0.96–1.01), *p* = 0.123 and HR 0.91 (95% CI 0.55–1.50), *p* = 0.702, respectively).

### Secondary outcomes

Beside localised infections not specifically related to the MCS, such as urinary tract infections and pneumonias, the three most commonly encountered adverse events were major bleeding, ventricular tachycardia and minor haemolysis with corresponding event rates of 0.51, 0.35 and 0.26 per patient year, respectively, as shown in Tab. 3.

**Table 3:** Complications (event rate per patient year) for the total cohort (*n* = 268)

Clinical data			Complications		
				Events	Event rate
Patient years—total	510	Cardiac arrhythmia—SVT		129	0.25
Patient years—HM-II	380	Cardiac arrhythmia—VT		180	0.35
Patient years—HVAD	99	Device malfunction—major		50	0.1
Patient years—HM 3	30	Device malfunction—minor		83	0.16
30-day mortality (%)	7.8	Haemolysis—major		76	0.15
90-day mortality (%)	11.2	Haemolysis—minor		131	0.26
Hospitalisation (days, mean ± SD)	50 ± 36	Hepatic dysfunction		68	0.13
*Postoperative data*		Hypertension		8	0.02
ICU stay (days, mean ± SD)	11 ± 12	Major bleeding—ENT		15	0.03
Ventilator (days, mean ± SD)	5.5 ± 9.7	Major bleeding—GI		72	0.14
Inotropics (days, mean ± SD)	5.8 ± 7.2	Major bleeding—other		174	0.34
		Major infection—exit site		82	0.16
		Major infection—pocket		15	0.03
		Major infection—sepsis		103	0.2
		Haemorrhagic stroke		25	0.05
		Ischaemic stroke		51	0.1
		Neurological dysfunction—TIA		30	0.06
		Pericardial fluid effusion		41	0.08
		Renal dysfunction—acute		50	0.1
		Renal dysfunction—chronic		4	0.01
		Respiratory failure		76	0.15
		Right heart failure		116	0.23

*SVT* supraventricular tachycardia, *VT* ventricular tachycardia, *HM-II* HeartMate II, *HM* *3* HeartMate 3, *major bleeding—ENT* major bleeding in the ear-nose-throat region, *major bleeding—GI* major gastro-intestinal bleeding, *TIA* transient ischaemic attack

Strokes (haemorrhagic and/or ischaemic) occurred 0.15 times per patient year. RHF occurred 0.23 times per patient year, most often (65%) within the first month after implantation. In 29 patients, RHF developed beyond 30 days after implantation, of whom 8 (28%) also suffered from early RHF.

## Discussion

This analysis of 268 patients, resulting in clinical experience of 510 patient years, describes the 5‑year outcome of cf-LVAD patients in a Dutch population, in whom the device was initially implanted as a bridge to transplantation. Survival at 1, 3 and 5 years was 83%, 72% and 57%, respectively, in this selected group of end-stage HF patients. This denotes its use as an extended bridge to heart transplantation, although still with considerable morbidity.

### Interpretation of findings

Previously, only a few smaller single-centre studies were performed regarding long-term results of cf-LVAD support. Takeda et al. presented their results in 140 patients, showing a survival rate of 83%, 75% and 61% after 1, 3 and 5 years, respectively [17]. We now confirmed these results in a larger population. In the most recent annual INTERMACS report, survival rates at 1, 3 and 5 years were 83%, 63% and 46%, respectively [18]. With regard to the pre-operative condition, it is known that patients in INTERMACS profiles 1–3 have worse survival rates, especially INTERMACS profile 1 [15, 18]. Our study confirmed the relationship between the initial poor state and the trend towards worse survival of patients in INTERMACS profile 1, in comparison to INTERMACS profile 2 or 3, despite prior stabilisation on short-term MCS, although this was not statistically significant.

Generally, in MCS patient selection is of utmost importance for the outcome. Stewart et al. studied the use of the INTERMACS classification to identify ambulatory patients with advanced HF who may benefit from a cf-LVAD. In that study, patients in INTERMACS profile 4 had a higher mortality rate and needed MCS more often compared to patients in INTERMACS profile 5–7 [19]. In addition, the ROADMAP trial concluded that patients in INTERMACS 4 have better survival, functional capacity and improved quality of life when treated with a cf-LVAD in comparison to optimal medical management [20].

Furthermore, prediction of RHF is important, because this is related to worse survival. Recently, the EUROMACS-RHF risk score was developed, which can be used to predict early RHF [21]. Unfortunately, little is known about risk factors for late RHF, which needs further research in the setting of long-term MCS.

Technical improvements in the HM 3, using a magnetically levitating environment, revealed fewer haemocompatibility-related adverse events (e.g. pump thrombosis) in comparison to HM-II at 2 years, as concluded in the MOMENTUM 3 trial. However, the rate of bleeding events was comparable in both groups [22–24]. A personalised anticoagulation regimen could decrease the individual risk for bleeding and thrombosis. Furthermore, the risk for infection could be decreased by the use of cf-LVAD with smaller (or no) external components including the driveline, which is the most frequently encountered location for VAD-related infections.

### Strengths and limitations

This is the largest single-centre study reporting on 5‑year outcome in cf-LVAD patients in whom the device was implanted as a bridge to transplantation, reflecting the Dutch results of long-term MCS. Complications were recorded prospectively and systematically in a central database. Patient follow-up was complete in our own centre, minimising the risk of missing data.

However, the single-centre design may indicate that our results cannot be extrapolated directly to other centres. Furthermore, in nearly all patients the cf-LVAD was implanted as a bridge to transplantation. In general these patients appear to have a more favourable outcome in comparison to those receiving this device as destination therapy [15, 18, 25]. Finally it has to be realised that our results are mainly based on the HM-II LVAD, as almost 60% of patients received this device.

## Conclusion

In our experience, based on 268 cf-LVADs, the use of cf-LVADs for end-stage HF demonstrated a survival of 57% after 5 years, proving relatively good long-term results. These results support the use of such devices as an extended bridge to heart transplantation, necessitated by the shortage of donor hearts. However, several important adverse effects need to be tackled by further technical improvements. Also risk stratification before cf-LVAD implantation in individual patients is essential. Presently we are only on the verge of acquiring this knowledge [26, 27]. The assessment of a personal risk model could improve individualised therapy, for example the anticoagulation regimen, which is now generally the same for each type of device and for every patient.

## Caption Electronic Supplementary Material

Supplementary table 1. Characteristics of the total population and per timeframe (2006–2012 and 2013–2017)
