# Supplementary material for: Outcome of mechanical circulatory support at the University Medical Centre Utrecht
Source: Neth Heart J. 2020 Feb 24;28(4):210–8. doi: 10.1007/s12471-020-01375-4 (PMC7113343; doi:10.1007/s12471-020-01375-4)
Supplement: Supplementary file 1 — Supplementary table 1. Characteristics of the total population and per timeframe (2006–2012 and 2013–2017) [file 12471_2020_1375_MOESM1_ESM.docx]

**Supplementary table 1. Characteristics of the total population and per timeframe (2006-2012 and 2013-2017)**

|  | **Total** | **2006-2012** | **2013-2017** | **p-value** |
| --- | --- | --- | --- | --- |
|  | N (%) | N (%)  109 (41)  47 (13)  77 (71)  59 (54)  31 (28)  2 (2)  7 (6)  3 (3)  5 (5)  0 (0)  2 (2)  6 (6)  23 (21)  56 (51)  22 (20)  2 (2)  0 (0)  98 (90)  11 (10)  0 (0) | N (%)  159 (59)  53 (13)  108 (68)  90 (57)  39 (25)  3 (2)  4 (3)  0 (0)  2 (1)  1 (1)  20 (13)  4 (3)  29 (18)  56 (35)  49 (31)  20 (13)  1 (1)  61 (38)  60 (38)  38 (24) |  |
| **Total** | 268 (100) |  |  |  |
| **Age** (Mean ± SD) | 50 (13) |  |  | < 0.001 |
| **Gender**–male | 185 (69) |  |  | 0.636 |
| **Etiology of cardiomyopathy** | 146 (54.5)  5 (1.9)  69 (25.7)  11 (4.1)  3 (1.1)  7 (2.6)  1 (0.4)  26 (9.7)  10 (3.7)  52 (19.4)  112 (41.8)  71 (26.5)  22 (8.2)  1 (0.4)  159 (59.3)  71 (26.5)  38 (14.2) |  |  |  |
| Dilated |  |  |  | 0.689 |
| Hypertrophic |  |  |  | 0.474 |
| Ischemic |  |  |  | 0.672 |
| Myocarditis |  |  |  | 0.128 |
| Peri-partum |  |  |  | 0.066 |
| Toxic |  |  |  | 0.124 |
| Congenital |  |  |  | 1.000 |
| Other |  |  |  | 0.002 |
| **INTERMACS profile** |  |  |  |  |
| 1 Critical cardiogenic shock without MCS |  |  |  | 0.561 |
| 1* Critical cardiogenic shock with MCS |  |  |  | 0.325 |
| 2 Progressive decline on inotropic support |  |  |  | 0.008 |
| 3 Stable but inotrope dependent |  |  |  | 0.053 |
| 4 Resting symptoms home on oral therapy |  |  |  | 0.002 |
| 6 Exertion limited |  |  |  | 1.000 |
| **Device** |  |  |  |  |
| HM-II |  |  |  | <0.001 |
| HVAD |  |  |  | <0.001 |
| HM 3 |  |  |  | <0.001 |

^MCS=mechanical circulatory support; HMII= HeartMate II; HM3= HeartMate 3.^
